# Primary Ovarian Large B-Cell Lymphoma

**DOI:** 10.1155/2013/493836

**Published:** 2013-10-07

**Authors:** Mine Islimye Taskın, Levent Gokgozoglu, Bedrı Kandemır

**Affiliations:** ^1^Department of Obstetrics and Gynecology, Balıkesir University School of Medicine, Balikesir, Turkey; ^2^Department of Obstetrics and Gynecology, Giresun Bulancak Puclic Hospital, Giresun, Turkey; ^3^Department of Pathology, Samsun Medical Park Hospital, Samsun, Turkey

## Abstract

The involvement of the ovary by malignant lymphoma is a well-known late manifestation of disseminated nodal disease. Primary ovarian lymphoma is rare. We herein describe a case of primary ovarian diffuse large B-cell lymphoma involving unilateral ovary in a 38-year-old woman which was detected incidentally. Preoperative ultrasonic imaging showed a 46∗42 mm heterogeneous cystic mass. Laparotomy revealed that left adnexal mass and left salpingo-oophorectomy was performed. The current diagnosis was determined after immunostaining. The patient was treated with R-CHOP regimen after the operation. She remains cancer-free 24 months after chemotherapy.

## 1. Introduction

 Lymphomas presenting as ovarian tumors are uncommon and may occur as de novo or secondary as a part of systemic disease [1]. Primary ovarian non-Hodgkin's lymphoma (NHL) is an extremely rare disease as other primary lymphomas of the genital tract, accounting for 0.5% of all NHLs and 1.5% of all malignant ovarian neoplasms [2]. It is considered that primary ovarian NHL arises from hilar lymphoid tissue or teratoma in the ovary. Fox et al. have suggested three criteria for the diagnosis of primary ovarian lymphoma: (1) tumor has confined to the ovary regional lymph nodes or adjunctive organs at the time of the diagnosis, (2) bone marrow and peripheral blood have not contained any abnormal cells, and (3) if extraovarian disease appear later, there must be a few months between the time of ovarian and extra-ovarian lesions [3]. The most common type of lymphoma involving the ovary is diffuse large B-cell lymphoma [4]. In this case we present a case of primary ovarian large B-cell lymphoma, which was diagnosed using immunohistochemistry.

## 2. Case Report

 A 38-year-old woman, gravida 4, para 4, was admitted to the hospital with complaints of irregular bleeding from the vagina for two-month duration, and she also had a desire of a tubal ligation. A physical examination, pelvic exam, and digital rectal examination were normal. Routine hematology and chemistry showed no abnormality. Serum carcinoembryonic antigen, cancer antigen 125, and cancer antigen 19–9 were within normal limits. Cancer antigen 15–3 was positive 30, 33 (normal range 0–25). Ultrason examination showed a 46∗42 mm heterogeneous cystic mass surrounded by thick wall and containing intense fluid in the left ovary whereas the right ovary was normal. Because of the ultrasonographic appearance and cancer antigen levels, this cystic mass was evaluated as benign and no further investigation was done.

 Laparatomy was performed. The patient was explored with a pfannenstiel incision. Laparatomy revealed that left adnexal mass measuring 5 cm was coarse and consisting of solid component. So left salpingo oophorectomy was performed. Peritoneal surface was clean and there was no ascetic fluid accumulation. Right ovary and fallopian tube were normal.

 The histology of the specimen was malignant but suspicious for undifferentiated carcinoma. The definite pathologic diagnosis after immunostaining was diffuse, large-cell malignant lymphoma of B-cell lineage. Work-up for the lymphoma after the surgery included abdominal and thoracic magnetic resonance imaging, bone marrow biopsy, and a whole body positron emission tomography scan. The findings from all these studies showed no other sites involved. The patient later received an adjuvant R-CHOP chemotherapy (rituximab, 375 mg/m^2^ day; cyclophosphamide, 750 mg/m^2^ day; doxorubicin, 50 mg/m^2^ day; vincristine, 1.4 mg/m^2^ day; prednisone, 50 mg/m^2^ day) 6 times intravenously and treatment progressed well. The patient is alive without disease 24 months after the operation without additional surgery.

**Figure 1:**
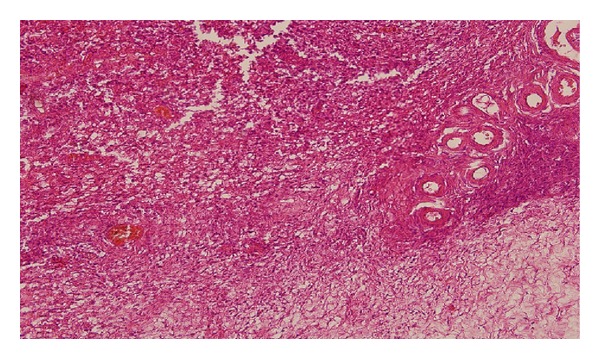
Tumor cells infiltrating the ovarian tissue. Diffuse growth pattern of tumor cells (H&E, ×10 magnification).

 Microscopic examination revealed diffuse growth pattern of tumor cells with large vesicular nuclei and prominent nucleoli. Tumor cells consisted of diffuse large lymphocytes, had abundant cytoplasm, and were eosinophilic (Figure 1).

**Figure 2:**
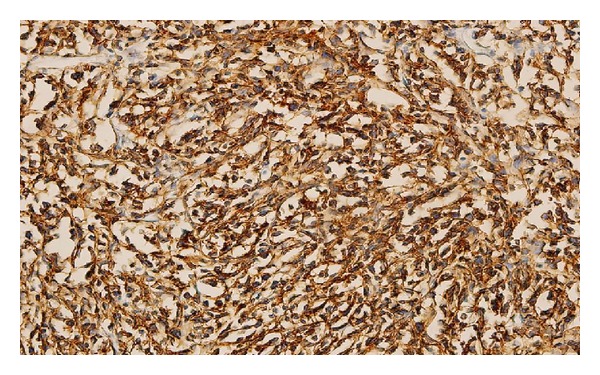
Paraffin immunostaining studies showed strong positivity of the neoplastic cells for B-cell CD20.

**Figure 3:**
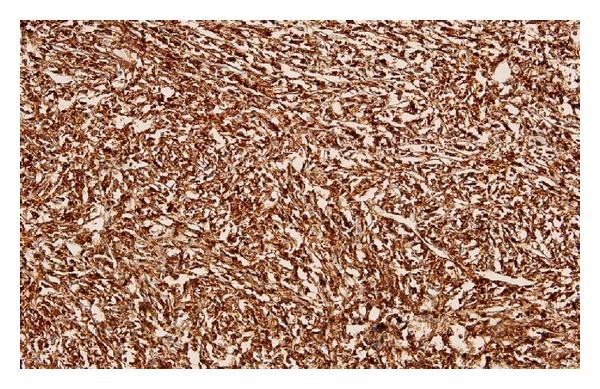
Paraffin immunostaining studies showed strong positivity of the neoplastic cells for leucocyte commen antigen (LCA).

**Figure 4:**
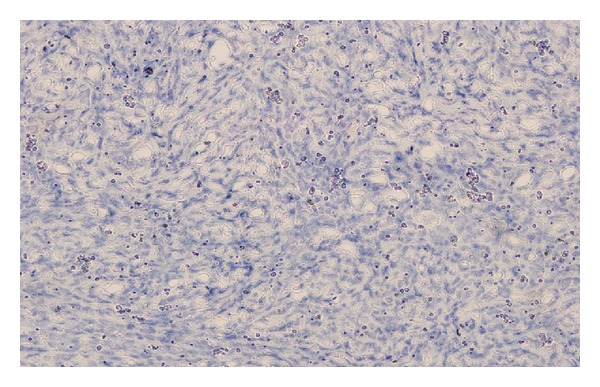
Immunostaining was negative for PLAP.

 Paraffin immunostaining studies showed strong positivity of the neoplastic cells for B cell CD20 and leucocyte commen antigen antibody (LCA) (Figures 2 and 3). Reactive T cell was stained with CD3. Tumor cells were negative for the T cell marker CD3. No immunoreactivity was noted with staining inhibin, CD117, and PLAP (Figure 4). These findings were in favor of diffuse large-cell malignant lymphoma of B-cell lineage.

## 3. Discussion 

 Non-Hodgkin lymphoma rarely involves the gynecologic tract. However, when involved, the ovary is one of the more commen anatomic sites [4]. True primary ovarian lymphomas are even rarer. Primary ovarian NHL accounts for 0.5% of extranodal NHL and 1.5% of primary ovarian cancers [2]. The most common histologic types involved in primary ovarian NHL are Burkitt lymphoma and diffuse large B-cell lymphoma [5]. The differential diagnosis of solid ovarian tumors includes rhabdomyosarcoma, extragonadal teratoma, neurogenic granulosa cell tumor, and disgerminoma, and definitive diagnosis can be only confirmed by pathologic examination of the tumor tissue. In our case in the first histologic evaluation the diagnosis was suspicious. Pathologist suspected lymphoma and germ cell tumor. Definite pathologic diagnosis was performed after immunostaining. 

 The presence of positive staining for leukocyte commen antigen distinguishes malignant lymphoma from nonlymphoid neoplasms. In our case tumor cells were positive for LCA and B cell CD20.

 The difference between primary and secondary lymphomas is important in terms of prognosis. The initial clinical manifestation of an occult nodal lymphoma as an ovarian mass is known as having a poor outcome with a survival rate ranging from 7% to 38% at 5 years. But primary ovarian lymphoma has better prognosis [6]. The histologic type is probably the most important prognostic factor, with B-cell tumors being associated with longer survival [7]. In our case, diffuse large B cell lymphoma of the ovary has a favorable outcome and the patient is alive without disease for 24 months.

 Primary ovarian lymphomas are staged as other extranodal NHLs (Ann Arbor staging system) [3]. Treatment of primary ovarian lymphomas is based on histology, type, and clinical staging. Patients with localized disease usually have better prognosis [8]. In our patient, lymphoma was localized the unilateral ovary. Bone marrow biopsy, PETscan, and abdominal and thoracic MRI were clear. Ann Arbor stage was stage 1E. After the operation and clinic evaluation our patient was treated with 6 cycles of R-COP regimen of chemoteraphy every 3 weeks. No recurrence could be detected during a followup of 24 months.

 The question of whether some ovarian lymphomas can be considered truly primary in the ovary and not merely a localized initial manifestation of a generalized disease cannot be answered yet. But Fox et al. [3] have suggested three criteria for the diagnosis of primary ovarian lymphoma. If stringent criteria of Fox et al. are applied, primary ovarian lymphoma becomes vanishingly rare. Our case was obviously primary ovarian malignant lymphoma, not a part of systemic disease. Because our case fulfilled all the criteria, we operated the patient for left ovarian cystic mass. There was no other pathologic findings about the pelvic organs. After the operation a whole body screening was clean.

Ferrozzi et al. [9] reported eight patients with ovarian NHL (two primary lymphomas and six systemic NHLs) and assessed their most typical imaging patterns. Ovarian lymphomas were frequently bilateral and homogeneous, without ascites, and the tumors always exceeded 5 cm in diameter. Ultrasonography showed homogeneous, hypoechoic, and mildly vascularized tumors. Ultrasonographic features are nonspecific. In our case ovarian mass was unilateral, heterogeneous, and cystic. In the operation it was seen that the solid component was clear. So salpingo oophorectomy was performed. Frozen section cannot be performed because of the conditions of the clinic. Patients with ovarian lymphomas are mostly diagnosed after surgery and are treated with chemotherapy [10] as in our case. Radiotherapy is optional.

Yamada et al. [7] report a case with a diffuse large B cell ovarian malignant lymphoma, which presented as an advanced carcinoma successfully treated with CHOP regimens. In this case R-CHOP regimen was used and well tolerated by the patient.

 To conclude, if stringent criteria are used primary ovarian malignant lymphoma is a very rare disease. It is diagnosed after the operation using immunohistochemistry and treated with chemoteraphy. The authors experienced a case of a primary ovarian lymphoma presenting a heterogeneous cystic mass. Patients with localized disease and B cell phenotype have a good prognosis.
